# Oriented Scanning Is the Leading Mechanism Underlying 5′ Splice Site Selection in Mammals

**DOI:** 10.1371/journal.pgen.0020138

**Published:** 2006-09-01

**Authors:** Keren Borensztajn, Marie-Laure Sobrier, Philippe Duquesnoy, Anne-Marie Fischer, Jacqueline Tapon-Bretaudière, Serge Amselem

**Affiliations:** 1 Faculté de Médecine, Université Paris-Descartes, INSERM U428, Paris, France; 2 Hôpital Henri-Mondor, INSERM U654, Créteil, France; RIKEN Genomic Sciences Center, Japan

## Abstract

Splice site selection is a key element of pre-mRNA splicing. Although it is known to involve specific recognition of short consensus sequences by the splicing machinery, the mechanisms by which 5′ splice sites are accurately identified remain controversial and incompletely resolved. The human *F7* gene contains in its seventh intron (IVS7) a 37-bp VNTR minisatellite whose first element spans the exon7–IVS7 boundary. As a consequence, the IVS7 authentic donor splice site is followed by several cryptic splice sites identical in sequence, referred to as 5′ pseudo-sites, which normally remain silent. This region, therefore, provides a remarkable model to decipher the mechanism underlying 5′ splice site selection in mammals. We previously suggested a model for splice site selection that, in the presence of consecutive splice consensus sequences, would stimulate exclusively the selection of the most upstream 5′ splice site, rather than repressing the 3′ following pseudo-sites. In the present study, we provide experimental support to this hypothesis by using a mutational approach involving a panel of 50 mutant and wild-type *F7* constructs expressed in various cell types. We demonstrate that the *F7* IVS7 5′ pseudo-sites are functional, but do not compete with the authentic donor splice site. Moreover, we show that the selection of the 5′ splice site follows a scanning-type mechanism, precluding competition with other functional 5′ pseudo-sites available on immediate sequence context downstream of the activated one. In addition, 5′ pseudo-sites with an increased complementarity to U1snRNA up to 91% do not compete with the identified scanning mechanism. Altogether, these findings, which unveil a cell type–independent 5′−3′-oriented scanning process for accurate recognition of the authentic 5′ splice site, reconciliate apparently contradictory observations by establishing a hierarchy of competitiveness among the determinants involved in 5′ splice site selection.

## Synopsis

Typically, mammalian genes contain coding sequences (exons) separated by non-coding sequences (introns). Introns are removed during pre-mRNA splicing. The accurate recognition of introns during splicing is essential, as any abnormality in that process will generate abnormal mRNAs that can cause diseases. Understanding the mechanisms of accurate splice site selection is of prime interest to life scientists. Exon–intron borders (splice sites) are defined by short sequences that are poorly conserved. The strength of any splice sequence can be assessed by its degree of homology with a splice site consensus sequence. Within exons and introns, several sequences can match with this consensus as well as or better than the splice sites. Using a system in which a splice site sequence is repeated several times in the intron, the authors showed that linear 5′−3′ search is a leading mechanism underlying splice site selection. This scanning mechanism is cell type–independent, and only the most upstream splice site of all the series is selected, even if splice sites with a better match to the consensus are in the vicinity. These findings reconciliate contradictory observations and establish a hierarchy among the determinants involved in splice site selection.

## Introduction

A problem in mammalian pre-mRNA splicing is deciphering the mechanisms underlying the recognition of authentic signals for proper splicing. The accurate recognition of exons is a process that requires assembly of the major spliceosome, a macromolecular machinery that involves the coordinated action of small nuclear RNAs (snRNAs) and more than 100 polypeptides [1,2]. Any abnormality in that process will generate aberrant mRNAs that are either unstable or code for defective and/or deleterious protein isoforms [3]. As a puzzling paradox, in higher eukaryotes, introns are essentially defined by three short and poorly conserved sequences: the 5′ splice site, the branch point, and the 3′ splice site [4,5]. Moreover, as splice site consensus motifs are degenerated, within a typical mammalian transcript, several sequences, in addition to the authentic splicing elements, referred to as pseudo-sites, may match the consensus splice site signals, and sometimes even better than the real splice sites. These elements define a set of pseudo-exons that greatly outnumber genuine exons, but that are normally not included in mature mRNAs [6].

A number of structural features has been shown to play a key role in 5′ splice site selection [7]. One of them is the splice site sequence itself. The 5′ splice site consensus sequence comprises nine partially conserved nucleotides at the exon–intron boundary: MAG/guragu (with M and r standing for A or C and a or g, respectively, and / denoting the cleavage site). This consensus actually reflects the base pairing between the donor splice sequence and the 5′ terminus of the U1snRNA, which is involved in the early steps of splicing [8]. For a given splice site, the strength of this interaction is usually assessed by its consensus value (CV) [4]. Besides this interaction, different sets of auxiliary cis-regulatory elements, known as splicing enhancers or silencers, contribute to the identification of authentic 5′ splice sites [1].

The situation may be complicated by the presence, within several genes, of minisatellites, with their first monomer element spanning an exon–intron boundary. As a consequence, the exact sequence of the splice donor site is reiterated in the following intron. Such minisatellites provide an outstanding physiological model to study the mechanisms underlying splice site selection. However, the resulting splicing pattern has been investigated in very few cases only, and demonstrated the activation of only the most and second-most upstream pseudo-sites. Such is the case for the second intronic sequence of the human interferon-inducible *6–16* gene, in which the authentic splice donor site is followed by 25 pseudo-sites, whereas transcript analysis demonstrated the constitutive utilisation of the two most proximal pseudo-sites in addition to the authentic one [9]. Two pseudo-sites are also utilised in addition to the authentic splice donor site in the human *CBS* gene, encoding the cystathionine beta-synthase, that contains a minisatellite in intron (IVS) 13 with 15−20 monomer repeats[10,11]. In the human *LAMIN B2* gene, which contains a minisatellite consisting of 10−15 repeats of a 100-bp monomer, only the authentic splice donor site is used [12]; a similar situation has been observed in the *PDGF* gene that contains 16−18 repeats of an 81-bp monomer element in intron 4 [13]. In all these cases, the mechanism precluding the use of pseudo-sites has not been investigated.

Like the above-mentioned genes, the *F7* gene (GENBANK access: NM_000131), which encodes FVII (zymogen of a blood coagulation serine protease), displays a peculiar organization with a minisatellite located within IVS7 [14]. In IVS7, the first 37-bp monomer element consists of the last four bp of exon 7 and the first 33 bp of IVS7 [15]. This minisatellite is polymorphic, with at least five different alleles containing five to nine repeats of this 37-bp monomer ([16–19]). The IVS7 5′ authentic donor splice site UGG/gugggu (where / represents the cleavage site) is, therefore, followed by four to eight copies of 5′ pseudo-splice sites that are strictly identical in sequence; however, strikingly, in physiological conditions, only the most upstream 5′ splice site is used [15]. The study of *F7* pre-mRNA splicing thus provides an opportunity to assess the mechanism underlying 5′ splice site selection for transcripts of particular clinical importance, as judged by the potentially severe phenotype of patients with inherited FVII deficiency [20].

In previous work, we investigated the functional consequences of a transversion located within the authentic IVS7 splice donor site of the *F7* gene from a patient with severe FVII deficiency [21]. We showed that the mutation resulted in the activation of a single cryptic site corresponding to the 5′ pseudo-site located in the second IVS7 minisatellite monomer repeat. This observation led us to propose the existence of a physiological mechanism that, in the presence of consecutive splice consensus sequences, would exclusively stimulate and select the most upstream 5′ splice site, rather than repress the 3′ following pseudo-site(s) [21]. This model prompted us to investigate the mechanism underlying the accurate selection and activation of one particular 5′ splice donor site among five identical sites. In the present study, we address this issue using a mutational approach. Our findings unveil a nuclear 5′−3′-oriented scanning mechanism as the leading part of the splicing machinery, which is not cell type–dependent.

## Results

### *F7* IVS7 5′ Pseudo-Sites Are Potentially Functional, but Do Not Compete with the Authentic Donor Splice Site: Evidence for a Scanning Process in 5′ Splice Site Selection

**Figure 1:**
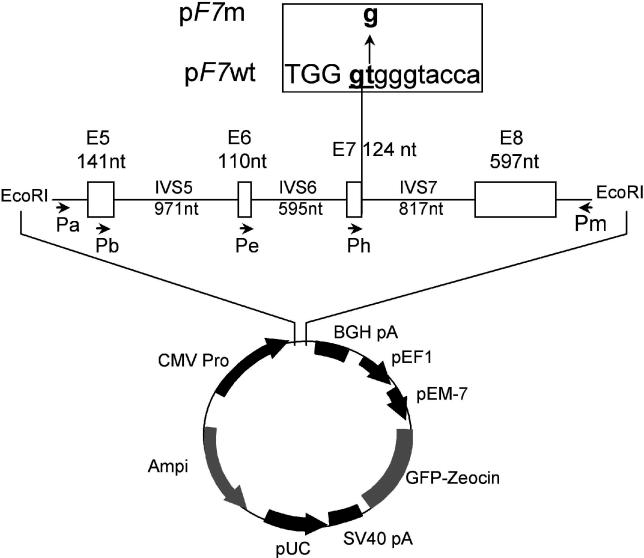
Schematic Representation of the Human *F7* Gene and Wild-Type and Mutant *F7* Minigenes Cloned into pTracer-CMV Vector Sizes of exons (E, open boxes) and introns (I, horizontal lines) are indicated. Primers Pa, Pb, Pe, Ph, and Pm are represented by horizontal arrows indicating their respective positions in the minigene sequence. Inset indicates the last three nucleotides of exon 7 (capital letters) and the first ten nucleotides of IVS7 (in lowercase characters) of the wild-type *F7* gene. The invariant gt dinucleotide is indicated in underlined bold letters. A vertical arrow indicates the nucleotide change in the mutant sequence.

**Figure 2:**
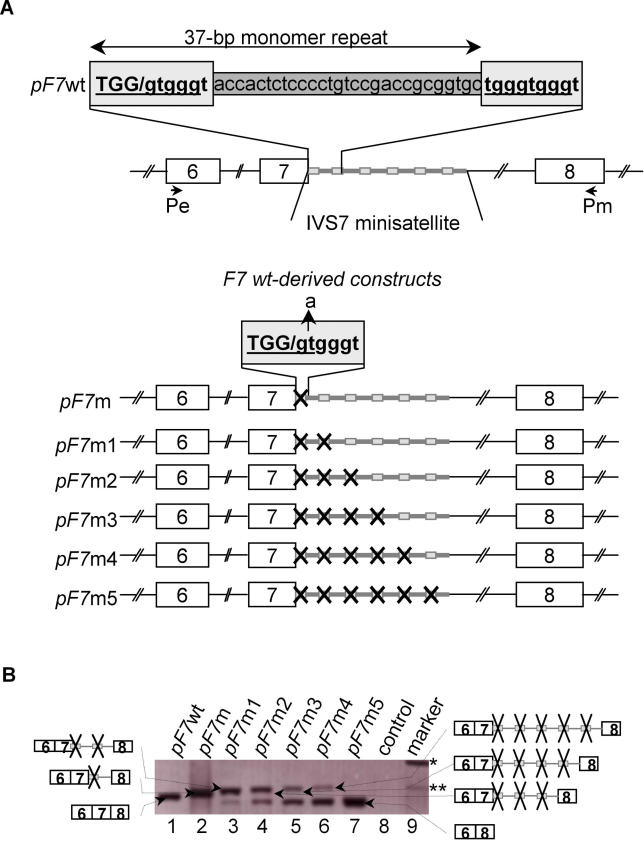
Assessment of the Ability of the IVS7 Pseudo-Sites to Be Activated (A) Schematic representation of the constructs used in this experiment. Top: Organization of the wild-type *F7* IVS7 proximal region (p*F7*wt); exons and introns are represented by open boxes and thin lines, respectively. The first 37-bp monomer, which spans the exon7–IVS7 boundary, is repeated within IVS7. The monomers are represented by a thick grey line. Each pseudo-splice site is represented by a grey box, and is separated from the next one by 28 bp. Bottom: Schematic representation of the various *F7* minigenes carrying a T-to-A transition located at the main dinucleotide of the consensus donor splice site and involving several consecutive 37-bp monomer elements found in IVS7. Mutations are represented by a black cross. (B) RT-PCR amplification of *F7* transcripts isolated from CHO cells transfected with the above-mentioned constructs. The products corresponding to the different PCR fragments, as identified by sequencing, are schematically represented on both sides of the figure.

To test whether each of the five IVS7 5′ pseudo-sites are potentially functional, in addition to the mutant minigene carrying the mutation identified in our patient with severe FVII deficiency and the wild-type construct (p*F7*m and p*F7*wt, Figures 1 and 2A), we generated five constructs (designated p*F7*m1 to p*F7*m5, see Materials and Methods and Figure 2A), with sequential inactivation of one to five pseudo-sites. RT-PCR amplification of *F7* transcripts isolated from Chinese hamster ovarian (CHO) cells transfected with the wild-type minigene yielded a 842-bp amplicon resulting from normal splicing of the entire IVS7 (Figure 2B, lane 1). This result, which was obtained with the use of primers Pe and Pm, was reproduced with other primer sets that flank IVS7 (unpublished data).

By contrast, in similar assays performed with the mutant constructs p*F7*m and p*F7*m1 to p*F7*m5 (Figure 2B, lanes 2–7), no normal splicing could be detected. More precisely, transient transfection of constructs p*F7*m1 to p*F7*m4 resulted in the generation of two kinds of aberrantly spliced products (Figure 2B, lanes 3–6), one of size that was always slightly larger than expected. Sequencing of the corresponding RT-PCR products demonstrated that sequential inactivation of the 5′ pseudo-sites led to the activation of the next, most upstream functional IVS7 5′ pseudo-site available. Such activation of a cryptic site led to the retention of short portions of IVS7 in the corresponding mutant cDNA between exons 7 and 8, accounting for the slight size differences between the resulting RT-PCR products (Figure 2B); sequencing of the second aberrantly spliced product, which was smaller in size than expected, showed that it corresponds to a unique molecular species lacking the entire exon 7 (exon skipping), thereby demonstrating that, for those constructs, the IVS6 donor splice site was utilized in combination with the IVS7 acceptor splice site. Strikingly, the relative amount of the two observed splicing products seemed to differ according to the construct, as shown after ethidium bromide staining (Figure 2B, lanes 3 to 6). Indeed, although our approach was not quantitative, the intensity of the larger products corresponding to the activation of each of the cryptic sites clearly decreased according to the number of inactivated pseudo-sites, while concurrently, the intensity of the smaller product lacking exon 7 increased. In keeping with these observations, in cells transfected with the p*F7*m5 construct, in which all pseudo-sites were inactivated, only the product resulting from exon 7 skipping could be detected (Figure 2B, lane 7), whereas in cells transfected with the p*F7*m construct, in which only the first 5′ splice site was inactivated, a single product was identified, resulting from the activation of the 5′ pseudo-site located in the second 37-bp monomer element (Figure 2B, lane 2). For all transfection experiments, examination of 50 additional clones obtained after subcloning of non-purified RT-PCR products generated with primers Pe and Pm failed to detect any other splice variants, thereby indicating that such species, if they indeed exist, are expressed at extremely low levels.

Altogether, these data show that the five IVS7 5′ pseudo-sites are potentially functional but do not compete for splicing with the IVS7 authentic donor splice site. They also reveal that when several adjacent pseudo-sites are inactivated, only the most upstream functional IVS7 5′ pseudo-site available is used, suggesting the existence of an accurate mechanism underlying the selection of the active 5′ splice site.

### Selection of the 5′ Splice Site Follows a Scanning-Type Mechanism Precluding Competition with Other Most Upstream Functional 5′ Pseudo-Site Available on Immediate Sequence Context

**Figure 3:**
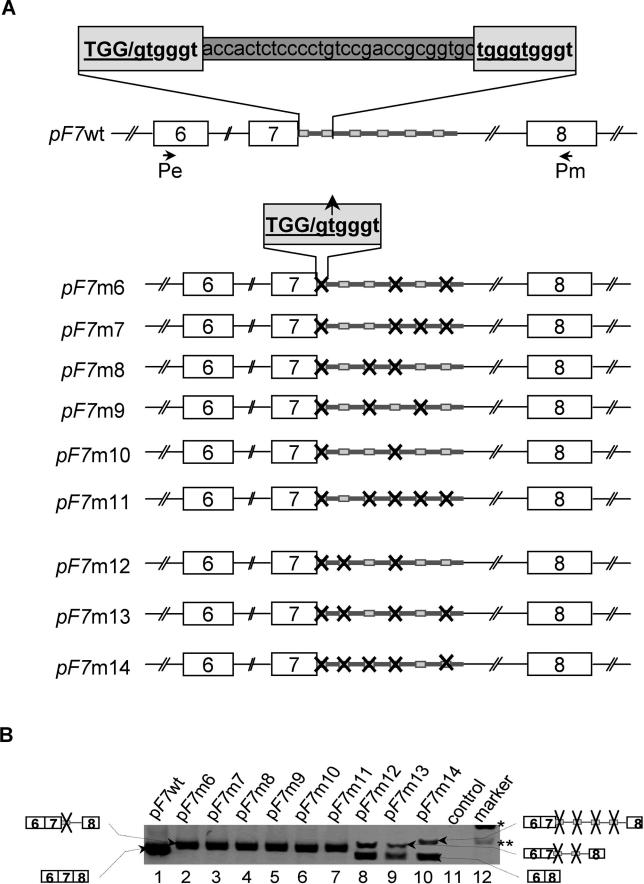
Splice Site Selection in the Context of Functional 5′ Pseudo-Sites, Separated by One or Several Inactivated 5′ Splice Sites (A) Schematic representation of the constructs used in this experiment (see legend to Figure 2A for the meaning of each symbol). (B) RT-PCR amplification of *F7* transcripts isolated from CHO cells transfected with the above-mentioned constructs. The products corresponding to the different PCR fragments, as identified by sequencing, are schematically represented on both sides of the figure.

To gain insights into the mechanism underlying selection of the donor splice site, we next generated *F7* minigenes containing functional 5′ pseudo-sites, separated by one or several inactivated 5′ splice sites (Figure 3A). As shown in Figure 3B, RT-PCR amplification of the transcripts expressed by CHO cells transfected with the mutant constructs p*F7*m6 to p*F7*m11—in which the first pseudo-site was always mutated—yielded a single band slightly larger in size than the one obtained with the wild-type construct (lanes 2 to 7); as shown after sequencing, this larger molecular species results from activation of the next functional 5′ pseudo-site.

To confirm the hypothesis that the most upstream 5′ splice site is selected for splicing, we studied the *F7* transcripts generated from CHO cells transfected with three additional constructs (p*F7*m12 to p*F7*m14) in which the first, second, and fourth pseudo-sites were inactivated in combination with either the third or the sixth pseudo-site (Figure 3A). As shown in Figure 3B, PCR amplification of the corresponding cDNAs resulted in the generation of two kinds of aberrantly spliced products (lanes 8 to 10): one of size always slightly larger than expected that resulted from the activation of the most upstream functional IVS7 5′ pseudo-site, and an additional band, smaller in size, that corresponded to a species in which exon 7 was skipped. Again, when two splice products were observed, their relative proportions seemed to differ according to the position of the available functional 5′ pseudo-site.

Taken together, these results are consistent with a scanning-type process of selection of the 5′ splice site, which precludes competition with other more downstream functional 5′ pseudo-sites. Indeed, other splicing mechanisms would predict the use of the first functional 5′ pseudo-sites downstream of the inactivated splice site, whereas such a scanning model predicts predominant use of the first available 5′ splice site.

### The Identified Scanning Mechanism for 5′ Splice Site Selection Is Not Cell Type–Dependent

**Figure 4:**
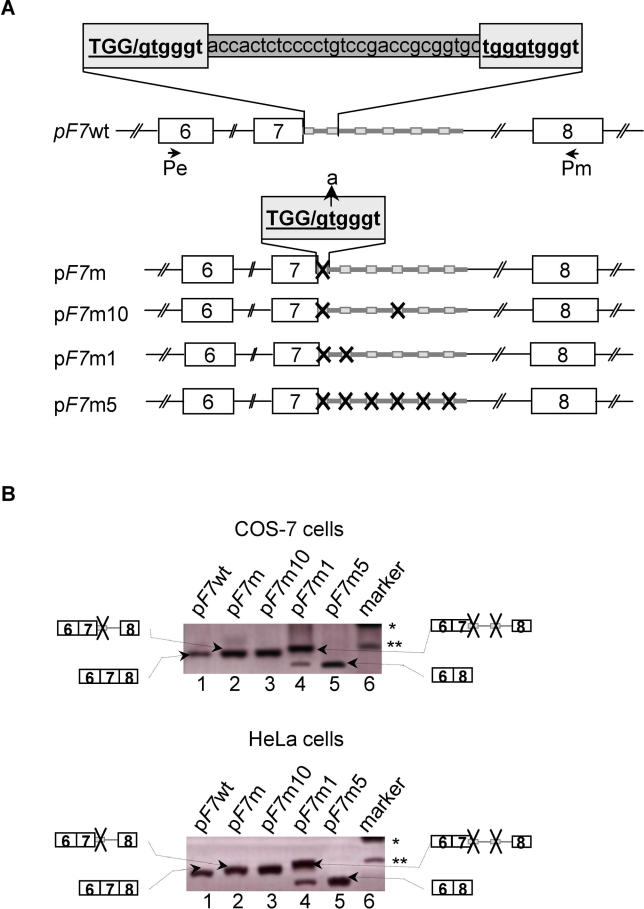
The Identified Scanning Mechanism for Splice Site Selection Is Not Cell Type–Dependent (A) Schematic representation of the constructs used in this experiment (see legend to Figure 2A for the meaning of each symbol). (B) RT-PCR amplification of *F7* transcripts isolated from COS-7 and HeLa cells transfected with the above-mentioned constructs. The products corresponding to the different PCR fragments, as identified by sequencing, are schematically represented on both sides of the figure.

The above-described experiments were performed in CHO cells. To test whether the identified scanning process is cell type–dependent, we performed similar studies in COS-7 and HeLa cells with *F7* minigenes that, when transfected in CHO cells, gave representative RT-PCR patterns: p*F7*m and p*F7*m10 that were associated with the use of the most upstream functional 5′ pseudo-site available (Figure 2B, lane 2, and Figure 3B, lane 6); p*F7*m12, associated with the same splicing event and exon skipping (Figure 3B, lane 8); and p*F7*m5 that was associated with exon skipping only (Figure 2B, lane 7). As shown in Figure 4, transfection of these minigenes in COS-7 and HeLa cells led to splicing patterns similar to those observed in CHO cells. These results provide evidence for a scanning mechanism that is not cell type–dependent, and, therefore, strongly suggest that the machinery involved in 5′ splice site selection is ubiquitous.

### 5′ Pseudo-Sites with a CV as High as 91 Do Not Compete with the Identified Scanning Process

**Figure 5:**
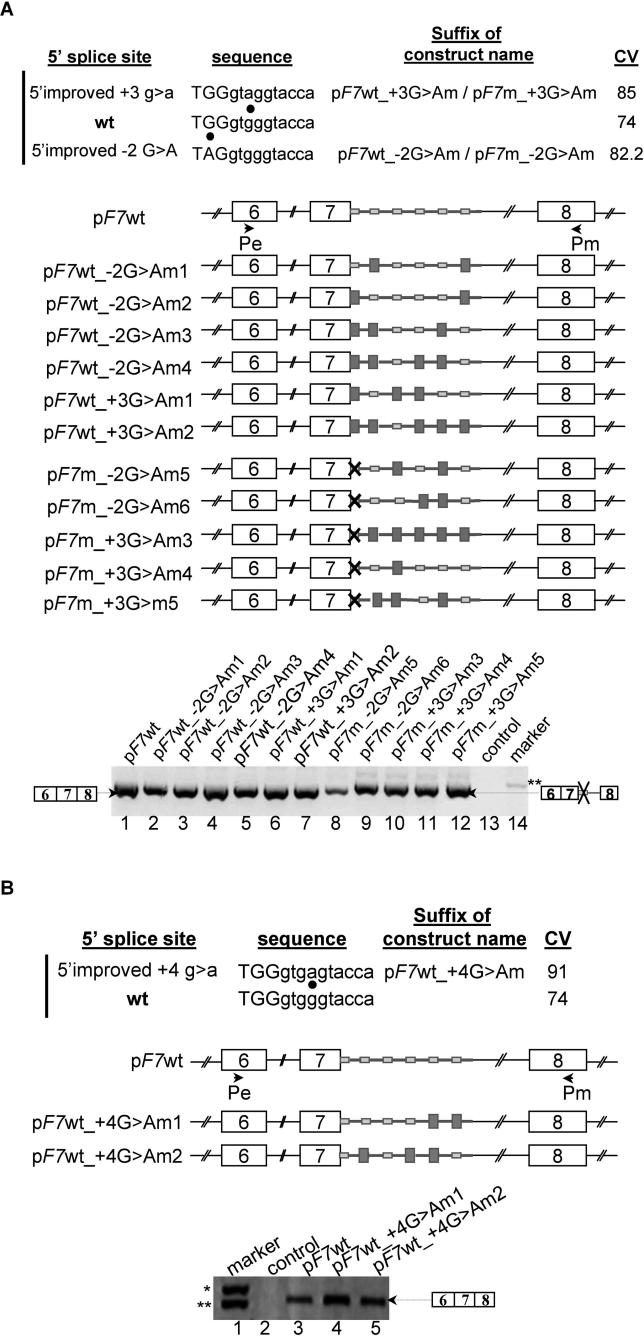
Splicing of a Series of Mutants with Increased Complementarity of the IVS7 5′ Pseudo-Sites to U1snRNA (A) Splicing of *F7* transcripts generated from *F7* minigenes with a CV of 85 or 82.2. (B) Splicing of *F7* transcripts generated from *F7* minigenes with a CV of 91. (A and B) Top: Wild-type (middle line in [A], lower line in [B]) and mutated (lower and upper lines in [A], upper line in [B]) 5′ pseudo-sites in the *F7* minigene. Dots show the location of the introduced mutations. Suffixes of the constructs' names and the resulting CVs are indicated. Middle: Schematic representation of the constructs transfected in CHO cells. Improved splice sites are represented by a hatched square (see legend to Figure 2A for the meaning of each symbol). Bottom: RT-PCR amplification of *F7* transcripts isolated from CHO cells transfected with the above-mentioned constructs. The products corresponding to the different PCR fragments, as identified by sequencing, are schematically represented on both sides of the figure.

**Figure 6:**
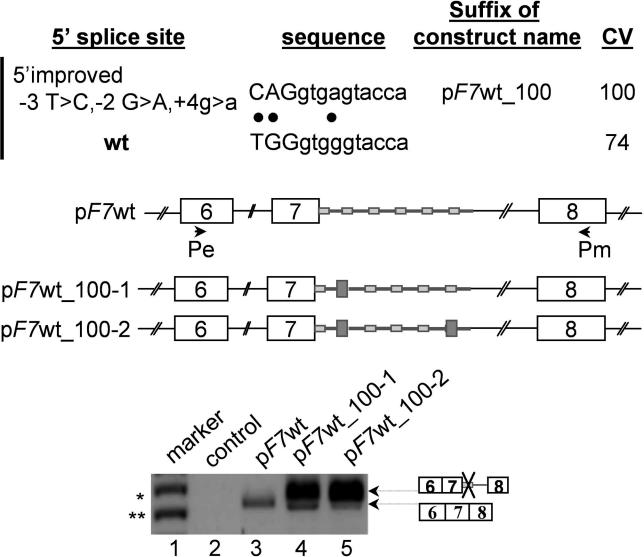
Splicing of a Series of Mutants with IVS7 5′ Pseudo-Sites Perfectly Matching U1snRNA Top: Wild-type (lower line) and mutated (upper line) 5′ pseudo-sites in the *F7* minigene. Dots show the location of the introduced mutations. Suffix of the constructs' names and the resulting CV of 100 are indicated. Middle: Schematic representation of the constructs transfected in CHO cells. Splice sites matching the consensus are represented by a hatched square (see legend to Figure 2A for the meaning of each symbol). Bottom: RT-PCR amplification of *F7* transcripts isolated from CHO cells transfected with the above-mentioned constructs. The products corresponding to the different PCR fragments, as identified by sequencing, are schematically represented on both sides of the figure.

The wild-type 5′ splice donor site sequence of IVS7 of the human *F7* gene (TGG/gtgggt), like each of the five 5′ pseudo-sites, offers a reasonable agreement with the consensus sequence of the splice donor sites, with a CV of 74 (Figure 5A), as assessed according to Shapiro and Senapathy [4]. To test whether 5′ pseudo-sites with higher CVs could compete with the identified scanning process, we transfected CHO cells with a series of *F7* minigenes carrying different combinations of 5′ pseudo-sites with increased CV on a p*F7*wt or a p*F7*m background (Figures 5 and 6). The following mutations were introduced in order to increase the complementarity of the resulting transcripts with the 5′ end of the U1snRNA sequence: a G-to-A transition at position −2 or +3 of the TGG/gtgggt splice site (i.e., TAG/gtgggt or TGG/gtaggt with a CV of 82.2 or 85, respectively; see Figure 5A, top), and a G-to-A transition at position +4 of the TGG/gtgggt splice site (i.e., TGG/gtgagt with a CV of 91; see Figure 5B, top). As shown in Figure 5A and 5B (bottom), the PCR products generated from each construct demonstrated the exclusive use of the most upstream functional 5′ site, regardless of its CV (i.e., 74, 82.2, 85, or 91). These results were confirmed by subcloning and subsequent sequencing of the non-purified RT-PCR products.

We next tested whether the most upstream functional 5′ site is still used when it is followed by sequences matching a perfect splice donor site. To this end, we designed mutant *F7* minigenes carrying the following mutations that were introduced in the splice site sequences in order to match perfectly with the 5′ end of the U1snRNA sequence, in the context of p*F7*wt: a T-to-C transition at position −3, a G-to-A transition at position −2, and a G-to-A transition at position +4 of the TGG/gtgggt splice site (i.e., CAG/gtgagt with a CV of 100; see Figure 6, top). As shown in Figure 6 (bottom), two molecular species were generated from these latest constructs. Although noteworthy, with such constructs one would expect the exclusive use of the perfect sites; this experiment and the subsequent sequencing of those products showed that the optimal splice sequences were used with an efficiency of approximately 70% (p*F7*wt_100–1) and 90% (p*F7*wt*_*100–2), thereby demonstrating that the most upstream 5′ site is always used, even in the presence of a perfect splice site.

Taken together, these results show firstly that, even when the CV is as high as 91%, the 5′ pseudo-sites do not compete with the identified scanning process. Secondly, competition occurs only when those sequences match a perfect consensus (CV of 100), and even in that case, the scanning process is still active, as demonstrated by the use of the most upstream functional 5′ site.

### No Autonomous Regulating Element Lies in the Neighbouring Intronic Sequence

**Figure 7:**
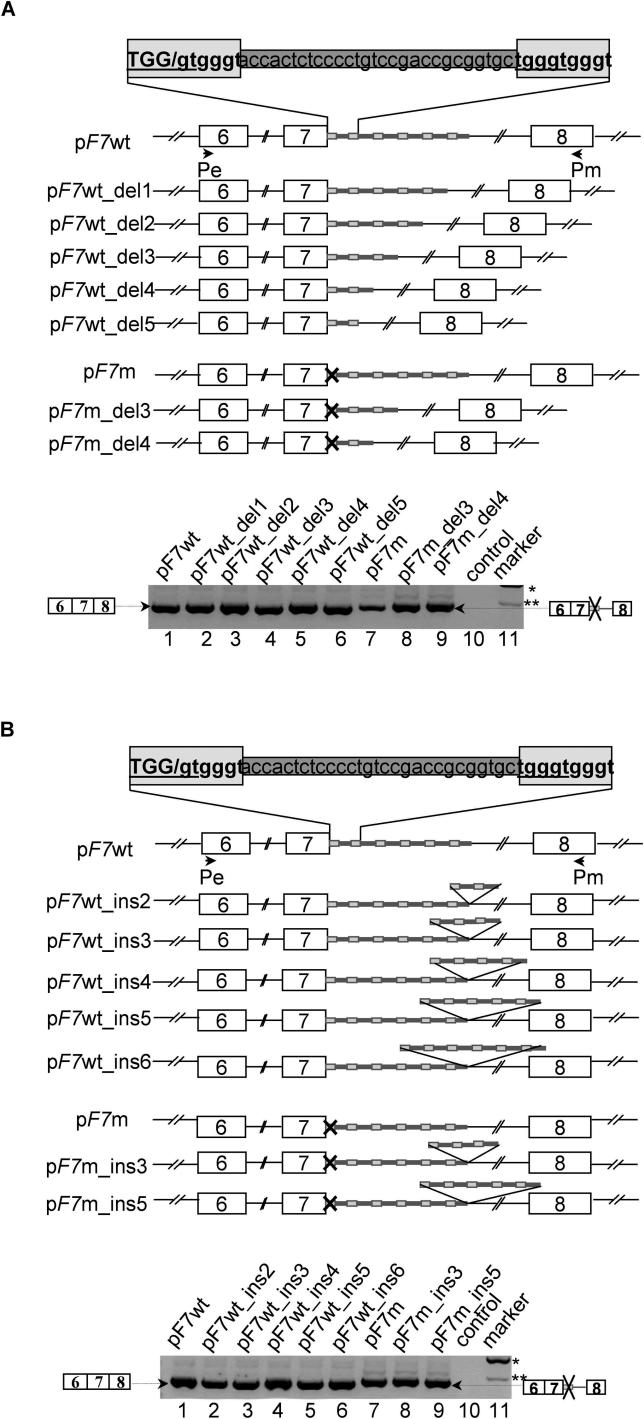
Search for Putative Intronic Sequences Regulating Splice Site Recognition (A) Top: Schematic representation of the constructs used in this experiment with deletions involving one or several 37-bp monomers in the context of *pF7wt* or *pF7m* (see legend to Figure 2A for the meaning of each symbol). Bottom: RT-PCR amplification of *F7* transcripts isolated from CHO cells transfected with the above-mentioned constructs. The products corresponding to the different PCR fragments, as identified by sequencing, are schematically represented on both sides of the figure. (B) Top: Schematic representation of the constructs used in this experiment with insertions of several 37-bp monomers in the context of *pF7wt* or *pF7m* (see legend to Figure 2A for the meaning of each symbol). Bottom: RT-PCR amplification of *F7* transcripts isolated from CHO cells transfected with the above-mentioned constructs. The products corresponding to the different PCR fragments, as identified by sequencing, are schematically represented on both sides of the figure.

Intronic splice enhancer sequences, when placed between competing 5′ splice sites, have been shown to favour the use of the upstream, most distal, 5′ splice site [22,23]; we, therefore, hypothesized that an intronic regulatory element, which could overlap one or several 37-bp monomer repeats, may either prevent the use of pseudo-sites or stimulate the use of the most upstream one. To test this hypothesis, we performed sequential deletions of the 37-bp monomer repeats of p*F7*wt and/or p*F7*m; the resulting constructs carrying one to six monomer repeats are shown in Figure 7A (top). As shown in Figure 7A (bottom), such truncations did not change the splicing pattern observed with the p*F7*wt- and the p*F7*m-derived constructs, suggesting that the deleted regions did not contain any regulatory element.

Similarly, we designed *F7* expression plasmids with the insertion, in p*F7*wt and/or p*F7*m, of several 37-bp monomer elements; the resulting constructs carrying eight to 12 monomer repeats are shown in Figure 7B (top). As shown in Figure 7B (bottom), the choice of the 5′ site used for splicing was not modified by the number of 37-bp monomer repeats contained in those constructs; indeed, in all cases only the most upstream functional site was activated. We therefore concluded that the neighbouring intronic sequence does not contain an overlapping regulatory element.

**Figure 8:**
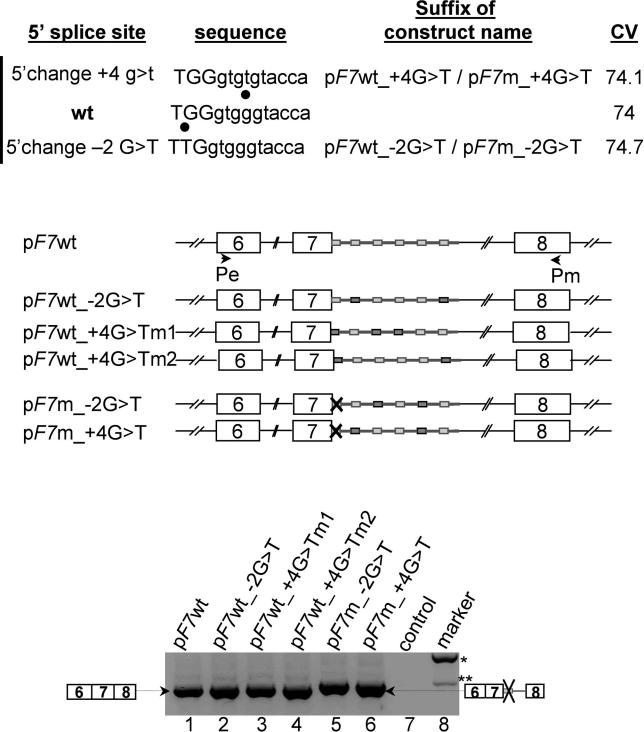
Role of G Triplets in IVS7 Splice Site Recognition Top: Wild-type (middle line) and mutated (lower and upper lines) 5′ pseudo-sites in the *F7* minigene. Dots show the location of the introduced mutations. Suffixes of the constructs' names and the resulting CVs are indicated. Middle: Schematic representation of the constructs transfected in CHO cells. Mutated splice sites are represented by a hatched box (see legend to Figure 2A for the meaning of each symbol). Bottom: RT-PCR amplification of *F7* transcripts isolated from CHO cells transfected with the above-mentioned constructs. The products corresponding to the different PCR fragments, as identified by sequencing, are schematically represented on both sides of the figure.

As for the 37-bp monomer repeats themselves, we noticed that they contain two G triplets that are small sequence elements known to modulate 5′ splice site selection in mammals [22,23] (see Figure 2A, in bold underlined letters)*.* To determine if these elements affect 5′ splice site selection, we modified their sequence, while keeping the CV of the corresponding 5′ splice sites at least equal to the CV of the wild-type sequence (74): a G-to-T transversion was introduced at position −2 or +4 of the TGG/gtgggt splice site (i.e., TTG/gtgggt or TGG/gtgtgt with a CV of 74.7 or 74.1, respectively; see Figure 8, bottom and middle). As shown in Figure 8 (bottom), mutations of the identified G triplets did not modify the splicing pattern, strongly arguing against their involvement in the selection of the most upstream 5′ splice site.

### The Scanning Process Initiates Downstream of Exonic Cryptic Sites

The “exon definition” model—based on the fact that, in vertebrates, exons are generally much smaller than introns—assumes that pairing between the splice sites occurs across an exon through the concerted recognition of the splice sites flanking this exon (reviewed in [5]). This model predicts that a mutation involving a 5′ splice site would prevent the recognition of the upstream exon, leading to skipping of this exon and/or activation of a cryptic site that lies in this exon.

**Figure 9:**
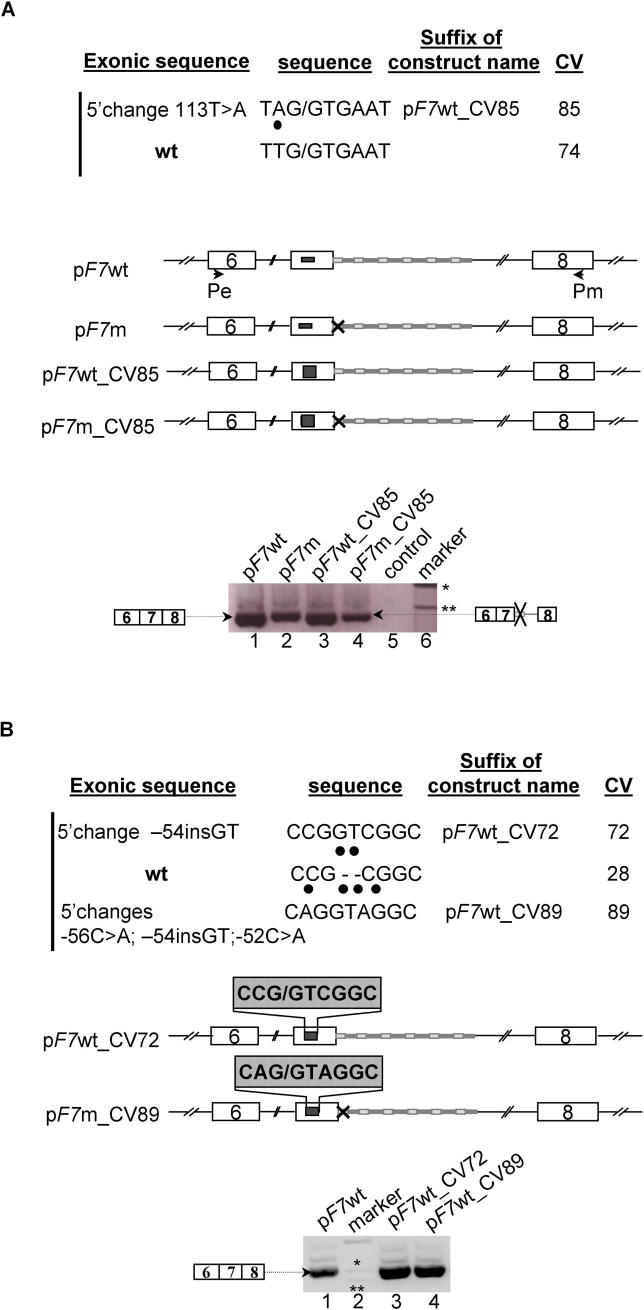
Effect on the Splice Site Selection of Exon 7 Sequences with Increased Complementarity to U1snRNA (A) Splicing of *F7* transcripts generated from *F7* minigenes harbouring an exonic cryptic site at −113 with a CV of 85. (B) Splicing of *F7* transcripts generated from *F7* minigenes harbouring an exonic cryptic site at −54 with a CV of 72 or 89. (A and B) Top: Wild-type (lower line in [A], middle line in [B]) and mutated (upper lines in [A], lower and upper line in [B]) 5′ pseudo-sites in the *F7* minigene. Dots show the location of the introduced mutations. Suffixes of the constructs' names and the resulting CVs are indicated. Middle: Schematic representation of the constructs transfected in CHO cells. Improved splice sites are represented by a hatched square (see legend to Figure 2A for the meaning of each symbol). Bottom: RT-PCR amplification of *F7* transcripts isolated from CHO cells transfected with the above-mentioned constructs. The products corresponding to the different PCR fragments, as identified by sequencing, are schematically represented on both sides of the figure.

To gain insight into the initiation step of the scanning process, we first looked for potential cryptic donor splice sites within exon 7 of the *F7* gene. As previously reported, a strong exonic cryptic site is located at IVS7–115, with a CV of 72, close to the CV of the authentic IVS7 5′ splice site sequence (CV of 74) [21]. We subsequently generated two constructs, derived from p*F7*wt and p*F7*m, in which this exonic cryptic site TTG/GTGAAT (CV of 72) was mutated toward TAG/GTGAAT (CV of 85), so that its resulting CV is above that of the 5′ authentic splice site (Figure 9A, top and middle), and tested whether this improved cryptic site could be selected for splicing. As shown in Figure 9A (bottom), this point mutation did not modify the splicing patterns, as compared with those associated with p*F7*wt and p*F7*m, a result that was confirmed by sequence analysis of the corresponding RT-PCR products. These results, therefore, support the hypothesis that, in these experimental conditions, the search for the active 5′ site initiates downstream of this exonic cryptic site*.*

However, an alternative explanation is the decreased splicing efficiency of unusually small exons: indeed, a 5′ splice site located too close to the 3′ splice site may have been ignored due to the excessively small size of the potential exon [24]. To test this hypothesis, we chose to work on another sequence located in exon 7 (CCGCGGC, with a CV of 28%) that, if selected for splicing, would yield an exon of 70 nucleotides (versus 124 nucleotides in the normal situation). This size seemed reasonable, since, as demonstrated recently, the length of abnormal exons that result from cryptic 5′ splice site usage is comprised of between 50 and 250 nucleotides [25]. We modified this sequence in order to generate two additional mutants harbouring the following potential splice sequences: CCGGTCGGC, with a CV of 72, very close to the CV of the authentic splice site (74), and CAGGTAGGC with a CV of 89, above that of the authentic 5′ splice site (Figure 9B, top and middle). Again, the PCR products generated from all the constructs and subsequent sequencing demonstrated the exclusive use of the authentic 5′ site (Figure 9B, bottom). These data strongly argue against an excessively small size of the potential exon as a structural feature precluding the use of an exonic cryptic donor splice site. Taken together, these results further strengthen the hypothesis that the scanning process initiates downstream of exonic cryptic sites.

## Discussion

The human *F7* gene, which encodes an essential coagulation factor, provides a remarkable model to decipher the mechanism underlying 5′ splice site selection in mammals. The first 37-bp monomer element of the polymorphic minisatellite located in IVS7 contains the exon7–IVS7 junction. As a consequence, in the primary transcripts, several IVS7 5′ splice sites (ranging from five to nine), identical in sequence, compete to join a single 3′ acceptor site. It is, however, noteworthy that physiologically, only the most upstream 5′ splice site is used, the unused 5′ splice sites being referred to as pseudo-sites. Therefore, IVS7 of the human *F7* gene must rely upon peculiar mechanisms to accurately control splice site selection. We have previously proposed a model imparting a strong preference for use of the most upstream 5′ pseudo-site, rather than some repression of the downstream following ones [21]. The results of the present study lend strong support to this model. They also provide several lines of evidence that favour an unbending scanning model that, in the context of several functional 5′ splice sites, would always select the most upstream 5′ splice site through a mechanism that precludes competition with other functional 5′ pseudo-sites*.*

This study was initiated to test whether the different 5′ pseudo-sites located in IVS7 of the *F7* gene are functional. Indeed, although all these sites are identical in sequence to the authentic one, one cannot exclude the possibility that they are unused because of a particular sequence context that could act through different means—such as, for instance, a secondary structure-dependent mechanism that would mask these pseudo-sites. We examined the possible secondary structure elements using different prediction of secondary structure tools, such as those at http://www.genebee.msu.su/services/rna2_reduced.html and http://www.bioinfo.rpi.edu/applications/mfold/rna/form1.cgi.

However, we did not show any arrangement specific to the sequence surrounding the pseudo-sites, which could explain the splicing phenotype observed in our study. Previous studies suggested that the 5′ splice sites whose activation would lead to a premature stop codon are not used so that an open reading frame is maintained [26,27]. Nevertheless, in our system, such a mechanism would predict the alternative use, in addition to the authentic 5′ splice site, of the fifth and sixth 5′ pseudo-sites that do not lead to a premature stop codon (unpublished data); as shown here, however, this is not the case. An alternative explanation accounting for the non-utilisation of the 5′ pseudo-sites is that, as suggested by others [22,23,28], they reside in a sequence context that hampers their utilisation. We could, however, exclude the involvement of intronic cis-acting auxiliary sequences in the splice site selection. Our data, which demonstrate that all 5′ pseudo-sites can be efficiently activated, argue against this hypothesis and rather suggest that these sites, though functional, are physiologically unused because of a competition-based mechanism. As shown here, the major determinant of this mechanism would be the location of these sites relative to each other: the more upstream intronic functional donor splice site is much more competitive and is actually the only one to be selected for splicing. These results strongly suggest a mechanism of oriented linear search for the selection of the 5′ splice site, starting upstream of the intervening sequence. Indeed, alternative modes of scanning processes would predict the use of other 5′ splice sites downstream.

The strength of this model is attested to by the fact that 5′ pseudo-sites with a CV as high as 91 still do not compete with the most upstream functional site, since they are not used for splicing, even at low efficiency. And only 5′ pseudo-sites with a CV of 100, hence perfectly matching the consensus sequence, can shift splicing toward their use, even though they still do not preclude the use of the most upstream functional site with a much lower CV. In this regard, it is important to underline that the optimal splice sequences are extremely rare in human genes. A compilation of authentic and cryptic donor splice sequences revealed that the average CV of authentic splice sites was 82.96 ± 6% [25], which is not only far below the optimal homology (CV of 100%), but also below the CV of pseudo-sites that we showed to be unable to compete with the most upstream 5′ splice. Moreover, a recent comparative analysis of human donor splice sequences showed that of 45,519 genes, only 2,360 contained a CV of 90% [29]. The low frequency of donor splice sequences perfectly matching the consensus could be explained by the fact that, as shown in Saccharomyces cerevisiae [30], hyperstabilization of the spliceosome (with more than seven potential Watson–Crick base pairs to U1snRNA) would inhibit the splicing process [31,32].

Our observations raise the larger question of the mechanism by which the most upstream functional site is recognised by the splicing machinery. In other words, what are the determinants involved in the initial step of such an oriented scanning process? This question is still open, although, in the case of *F7*, we could show that the increase of the CV of the exonic cryptic sites failed to induce their activation, thereby implying that scanning initiates downstream of these sites*.* Other data presented here, however, indicate that the extent to which the orientated scanning process is activated is distance-dependent: the further away the most upstream functional 5′ pseudo-site is from the authentic splice site, the more the IVS6 donor splice site is activated, leading to exon 7 skipping. These data, therefore, suggest that the identified oriented scanning process, even if predominant, is not exclusive. In this regard, other mechanisms such as those underlying exon definition [5], or those showing a decreased splicing efficiency of exons that are unusually large [33] or small [24], may also be involved in a balance that, in turn, favors one or the other 5′ splice site that flanks the defined exon, according to the distance in between these two sites.

Although a scanning process is widely accepted as an important mechanism for the selection of 3′ splice sites [34–39], none has gained general acceptance for the selection of the 5′ splice sites. Pioneering studies were performed using a model in which tandem duplications of the donor or acceptor RNA splice sites of the second intervening sequence were introduced in the human G gamma-globin gene. The transcript analysis demonstrated that splicing occurred only at the proximal copy of the duplicated splice sites [40,41]. From these results, in the presence of only two competing splice sites, a scanning model for splice site selection was proposed, but this model was still a matter of debate because a similar study performed by another group yielded opposite results [42]. The exon definition model also involves a scanning process; however, this one starts at the 3′ end of the upstream intron [5], and elements of the splicing complex then progressively scan the sequence downstream through the adjacent exon to locate a suitable 5′ splice consensus sequence. This model, therefore, predicts that a 5′ splice site mutation will lead either to the skipping of the exon upstream of the mutated 5′ splice site, or to activation of an exonic cryptic splice site that corresponds to the first suitable 5′ consensus sequence encountered by the spliceosome. Our data partly integrate the splicing patterns predicted by the exon definition model: as predicted by this model, we observed the skipping of exon 7 at different efficiencies for the constructs carrying consecutive inactivations of the more upstream pseudo-sites. However, they also diverge from this model in that activation of the strongest exonic cryptic donor splice site—as predicted by the exon definition model—was never observed, even when the CV of this cryptic site was increased, bringing further complexity to the characteristics of this mechanism. Finally, similar experiments performed in cells originating from different types and species, such as CHO, COS-7, and HeLa cells, gave rise to the same splicing patterns, providing evidence that the herein identified oriented scanning mechanism that occurs within this unusually arranged region does not require any cell-specific factor, but rather relies on the constitutive splicing machinery.

In summary, our results strongly suggest that, in the presence of consecutive potential splice sites in the neighbouring sequence, unbending scanning will rule the selection of the activated 5′ splice site. In the absence of such sequences, according to the above-mentioned studies, the selection of the activated splice site seems to rely upon its strength—i.e., its complementarity to U1snRNA. Taken together, our data reconciliate apparently contradictory observations in the mechanisms of 5′ splice site recognition in mammals by establishing a hierarchy of competitiveness among the determinants involved in this process.

## Materials and Methods

### Plasmid constructs.

A 3.5-kb *F7* genomic region spanning intron 4 to exon 8 (nt7688–11188) was amplified from a normal individual and from the FVII-deficient patient identified previously who carried a homozygous mutation (*9726T>G)* in IVS7 [21] The PCR amplification was performed with primers Pa and Pm (Figure 1) using the Expand high-fidelity PCR system (Boehringer Mannheim, Mannheim, Germany) according to the manufacturer's recommendations. Both the wild-type and the mutant F7 allele carried the six repeats of the 37-bp monomer element (b allele, unpublished data). The wild-type and mutant PCR fragments were cloned into the cytomegalovirus (CMV) promoter-based expression vector pTracer (Invitrogen, Carlsbad, California, United States) using the T4 DNA ligase (Invitrogen) according to standard procedures; the resulting constructs were designated p*F7*wt and p*F7*m, respectively.

These two expression plasmids were used as templates to generate 48 different constructs by site-directed mutagenesis using the QuickChange system (Stratagene, Amsterdam, Netherlands). The characteristics of the resulting plasmids are: the first series consists of 14 constructs—designated p*F7*m1 to p*F7*m14 (Figures 2 and 3)—carrying a T-to-A transition located at the main dinucleotide of the consensus donor splice site and involving several of the 37-bp monomer elements found in IVS7.

The second series of expression plasmids consists of 15 constructs: p*F7*wt_−2G>Am1 to p*F7*wt_−2G>Am4, p*F7*wt_+3G>Am1 and p*F7*wt_+3G>Am2, p*F7*wt_+4G>Am1 and p*F7*wt_+4G>Am2, and p*F7*wt_CV100–1 and p*F7*wt_CV100–2, which have been designed in order to improve the CV of one or several pseudo-sites in the context of p*F7*wt; and p*F7*m_−2G>Am5 and p*F7*m_−2G>Am6, and p*F7*m_+3G>Am3 to p*F7*m_+3G>Am5, which have been designed in order to improve the CV of one or several pseudo-sites in the context of p*F7*m (Figures 5 and 6).

The third series consists of 14 constructs (Figure 7): p*F7*wt_del1 to p*F7*wt_del5, which have been generated from p*F7*wt and carry a targeted deletion involving one to five of the 37-bp monomer elements found in IVS7, respectively. Similar deletions involving three or four of these monomer elements were generated from a p*F7*m background, thereby resulting in two expression plasmids designated p*F7*m_del3 and p*F7*m_del4. Constructs were also generated with insertion of a variable number of the 37-bp monomer element, either on a p*F7*wt background (i.e., p*F7*wt_ins2 to p*F7*wt_ins5) or from p*F7*m (i.e., p*F7*mins3 and p*F7*mins5).

The fourth series consists of five expression plasmids, which were designed to assess the functional importance of G triplets on splicing, were generated from p*F7*wt or p*F7*m and (Figure 8): p*F7*wt_−2G>T, p*F7*wt_+4G>Tm1, p*F7*wt_+4G>Tm2, p*F7*m_–2G>T, and p*F7*m_+4G>T.

The two following site-directed mutageneses were generated on p*F7*wt or p*F7*m in order to improve the CV (from 74 to 85) of a cryptic donor splice site located within exon 7 at position −113. The resulting plasmids were named p*F7*wt_CV85 and p*F7*m_CV85. The last two constructs, p*F7*wt*_*CV72 and p*F7*wt*_*CV89, have been designed in order to create a donor splice consensus sequence located within exon 7 at position −54 (Figure 9).

All primers used in amplification and mutagenesis steps are available on request. All the constructs were checked by sequencing the inserts and the vector flanking regions.

### Cell culture and transfections.

CHO cells were grown in ISCOVE medium (Invitrogen). HeLa cells and COS-7 cells were grown in DMEM medium. Both media were supplemented with 10% fetal calf serum (FCS) in a 5% carbon dioxide atmosphere at 37 °C. Transfections were performed at 60% of confluence by the Lipofectamin method (Invitrogen) in OptiMEM medium with 2 μg of each of the various constructs, according to the manufacturer's instructions. After 5 h, OptiMEM medium was removed and replaced by the normal medium supplemented with 10% FCS. Each transfection experiment was repeated at least twice.

### RNA isolation and cDNA synthesis.

Total RNA was extracted from the cells 48 h after transfection by using RNAplusTM (Bioprobe Systems, Montreuil-sous-bois, France) according to manufacturer's instructions. Following precipitation, RNA was treated with DNaseI to remove any contaminating plasmid DNA. First-strand cDNA synthesis was performed from 5 μg of total RNA, with random hexamers as primers (Pharmacia) and Superscript II (Invitrogen). One-tenth of each of the first-strand synthesis reaction products was used in each of the three subsequent PCR reactions with oligonucleotides Pb (5′ TCTGTGTGAACGAGAACGGC 3′) and Pm to amplify exons 5 to 8, Pe (5′ AAGAAATGCCAGCAAACCCC 3′) and Pm to amplify exons 6 to 8, and Ph (5′ GGAGCTCAGTTGTGTGGGGG 3′) and Pm to amplify exons 7 to 8 (see Figure 1). PCR amplifications were carried out using Expand high-fidelity PCR systems (Boehringer-Mannheim) as described above.

### Transcript analysis.

The PCR-amplified cDNA fragments were visualized after separation by electrophoresis on 1% agarose gel and revelation by ethidium bromide staining. Non-purified PCR products obtained in each reaction with the various constructs were subcloned into the plasmid vector pTOPO-4 (TOPO TA-Cloning, Invitrogen). For each subcloning experiment, at least 50 independent clones were sequenced on both strands in order to characterize the transcripts.

### CV analysis.

The statistical rules from Shapiro and Senapathy [4] were used to assign a score for the authentic and mutated IVS7 splice sites and the modified pseudo-sites, as previously described [21]. Briefly, these scores reflect the degree of conservation in different positions resulting from the alignment of 1,446 5′ splice sites of other genes. The consensus 5′ splice site sequence is MAG/guragu and spans from the position −3 (the third nucleotide from the 3′ end of the upstream exon) to +6 (the sixth nucleotide in the intron). A score of 100 represents the best match to the consensus, whereas 0 is the worst.

## Supporting Information

### Accession Numbers

The GenBank (http://www.ncbi.nlm.nih.gov/Genbank/index.html) accession number for the *F7* gene is NM_000131.

## Abbreviations

CHO: Chinese hamster ovarian
CV: consensus value
IVS7: intron 7 in human *F7* gene
snRNA: small nuclear RNA
